# Utility of 2.0 mm diameter Kirschner wires assembled with Wu’s Tension Traction Bows in calcaneal skeletal traction

**DOI:** 10.1038/s41598-024-67344-8

**Published:** 2024-07-16

**Authors:** Chaoqun Wang, Shengnan Dong, Xugui Li, Jiakai Ma, Ulrich Stoeckle, Tobias Gehlen, Zexi Ling

**Affiliations:** 1 Department of Traumatic Orthopedics, The Affiliated Hospital of Wuhan Sports University, No. 279 on Luoyu Road, Hongshan District, Wuhan City, 430079 Hubei Province China; 2 Center of Musculoskeletal Surgery, Charité Medical University Hospital Berlin, Augustenburgerplatz 1, 13353 Berlin, Germany

**Keywords:** Tibia fractures, Tension traction bow, Traction appliances, Skeletal traction, Preoperative management, Trauma, Clinical trial design, Musculoskeletal system

## Abstract

The 3.5 mm diameter or thicker Steinmann pins were commonly used in skeletal traction, which are so highly invasive that may result in severe complications such as pin tract infection and iatrogenic calcaneus fractures. Accordingly, Xirui Wu designed a new type of tension traction bow that can be assembled with 2.0 mm diameter Kirschner wires, but its effectiveness is unclear. We aim to evaluate the effectiveness of 2.0 mm diameter Kirschner wires assembled with Wu’s Tension Traction Bows in calcaneal skeletal traction. Data of 65 patients who were admitted to our department with tibia fractures from January 2021 to June 2022 and underwent preoperative calcaneal skeletal traction were collected retrospectively. 36 patients treated with 2.0 mm diameter Kirschner wires assembled with Wu’s Tension Traction Bows were assigned into Group 1, and 29 patients treated with 3.5 mm diameter Steinmann pins assembled with Bohler’s traction bows were assigned into Group 2. Pins loosening, breakage, and calcaneus fractures occurred in neither group. No statistical differences were observed in traction weight, swelling reduction efficacy, and traction duration (*P* > 0.05). Statistically significant differences were found between the two groups in term of post-traction bleeding incidence, average bleeding duration, and mean size and healing time of traction wounds (*P* < 0.05). Though VAS pain score before traction and on the first two days after traction in Group 1 didn't differ from Group 2 (*P* > 0.05), it was significantly lower in Group 1 compared to Group 2 on the third day after traction (*P* = 0.030). This study demonstrates that 2.0 mm diameter Kirschner wires assembled with Wu’s Tension Traction Bows produce satisfied traction outcomes with less invasion and are recommended in calcaneal skeletal traction.

## Introduction

In orthopedic clinical practice, skeletal traction is a crucial therapeutic method primarily utilized in patients with lower limb fractures or articular dislocations. This technique aims to prevent limb shortening and angular deformities following fractures, thereby preserving the alignment of the lower limb while simultaneously reducing swelling and pain^1–4^. Common forms of skeletal traction for lower limbs include supracondylar traction of the femur, traction of the tibial tubercle, and calcaneal traction. Calcaneal skeletal traction is often administered to patients with fractures in any part of the tibia or fibula. Traditionally, simple traction appliances with 3.5 mm diameter or thicker traction pins are employed to withstand effective traction forces and avoid bending or breakage. However, this may lead to post-traction bleeding at the pin sites, complicating post-traction nursing care. Additionally, healing time for traction wounds is typically prolonged after the removal of the traction pins, with occasional occurrences of iatrogenic calcaneus fractures associated with thick traction pins. Addressing the limitations of conventional traction devices, Xirui Wu^5^ devised a novel tension traction bow that could be assembled with 2.0 mm diameter Kirschner wires, capable of withstanding significant traction forces. However, the clinical efficacy of this innovation has yet to be systematically evaluated. In this retrospective study, we examined the clinical data of 65 patients with tibia fractures who underwent preoperative calcaneal skeletal traction using Wu’s Tension Traction Bows assembled with 2.0 mm diameter Kirschner wires. The objective of our investigation was to assess the clinical efficacy of these traction appliances and highlight their significance in the context of minimally invasive calcaneal skeletal traction techniques. By offering insight into the utilization of 2.0 mm diameter Kirschner wires with Wu’s Tension Traction Bows for calcaneal skeletal traction, this study contributes valuable information for enhancing patient care and promoting accelerated recovery following calcaneal skeletal traction.

## Material and methods

### Ethical statement

This study was approved by the Ethics Committee of the Affiliated Hospital of Wuhan Sports University, dated 01/29/2024, with number 672HREC20240117A. The informed consent in the format was obtained from the participants. The study was conducted in accordance with the Declaration of Helsinki.

### Patients

**Figure 1:**
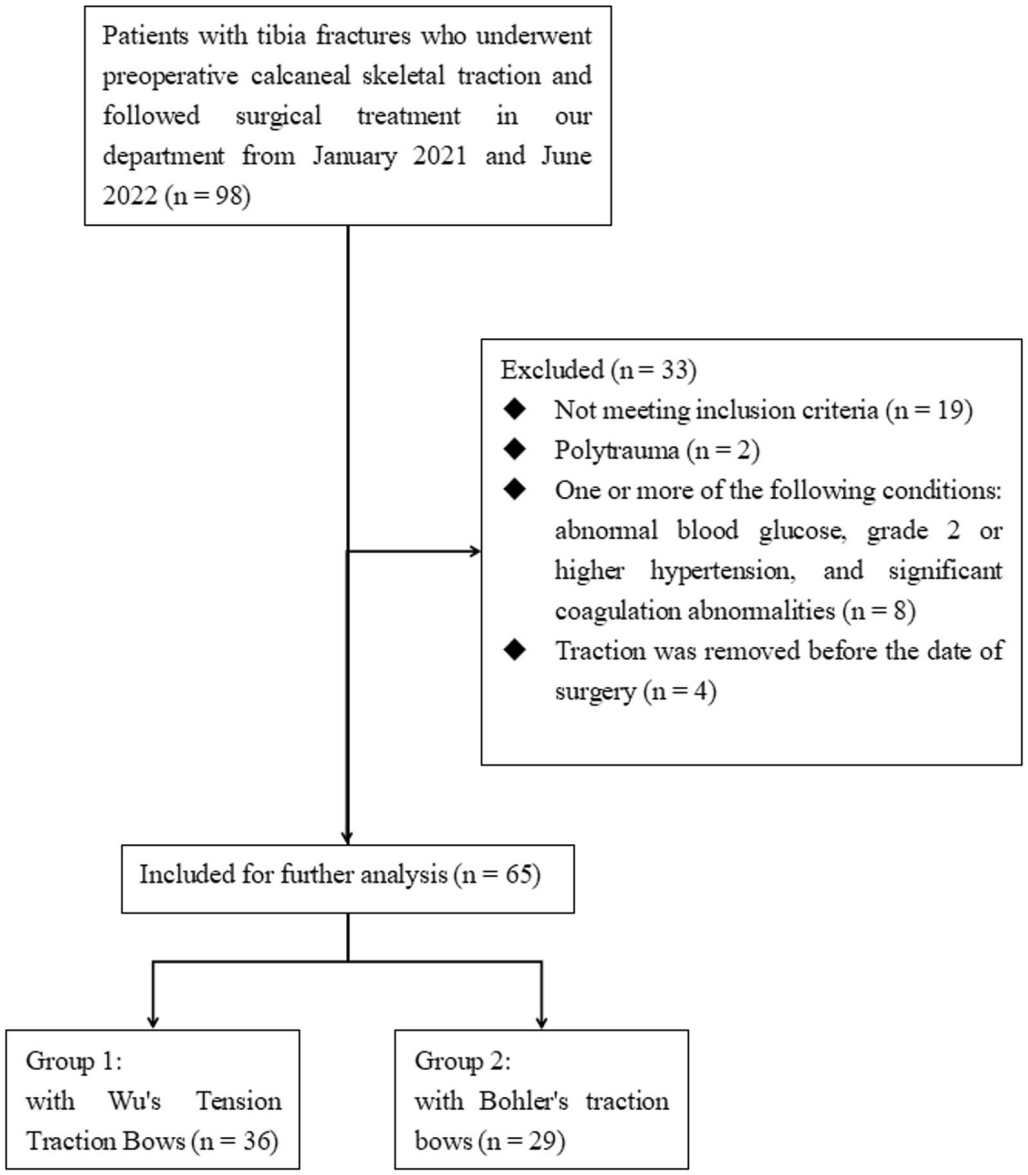
Study flowchart.

A retrospective study of patients with tibia fractures who underwent preoperative calcaneal skeletal traction and followed surgical treatment were performed in our department from January 2021 and June 2022. All fractures were caused by trauma. The inclusion criteria were as follows: (1) between the age of 18 and 65 years, (2) time from injury to admission < 72 h, (3) unilateral closed fractures of any part of tibia, (4) underwent surgical treatment, (5) underwent preoperative calcaneal skeletal traction, (6) previously in good health with no serious underlying medical conditions. Exclusion criteria included (1) polytrauma2, (2) abnormal blood glucose, (3) grade 2 or higher hypertension, (4) significant coagulation abnormalities, (5) skeletal traction was terminated in advance before the date of surgery. A total of 65 patients were identified in the Patient Document Archiving System (PDAS) database. The detailed inclusion and exclusion process is shown in Fig. 1. Based on different calcaneal skeletal traction appliances used, the selected cases were divided into 2 groups. Patients in Group 1 were treated with 2.0 mm diameter Kirschner wires assembled with Wu’s Tension Traction Bows. Patients in Group 2 were treated with 3.5 mm diameter Steinmann pins assembled with Bohler’s traction bows. The general information of participants was shown in Table 1.

**Table 1:** General information of participants in the study.

		Group 1 (n = 36)	Group 2 (n = 29)	*P* value*
Age (year)	Mean (SD)	38.3 (12.1)	40.1 (10.9)	0.529
Gender	Male	30	22	0.454
	Female	6	7	
Causing	Traffic accident	10	8	0.819
	Falling from height	16	11	
	Sprain	10	10	
Level of fracture	Proximal 1/3	15	12	0.619
	Middle 1/3	12	7	
	Distal 1/3	9	10	
Affected side	Left	16	13	0.975
	Right	20	16	

*A value of *P* < 0.05 was considered to be statistically significant.

### Traction procedures

Traction procedures for all patients in this study was performed by two resident surgeons with the same level of experience.

**Figure 2:**
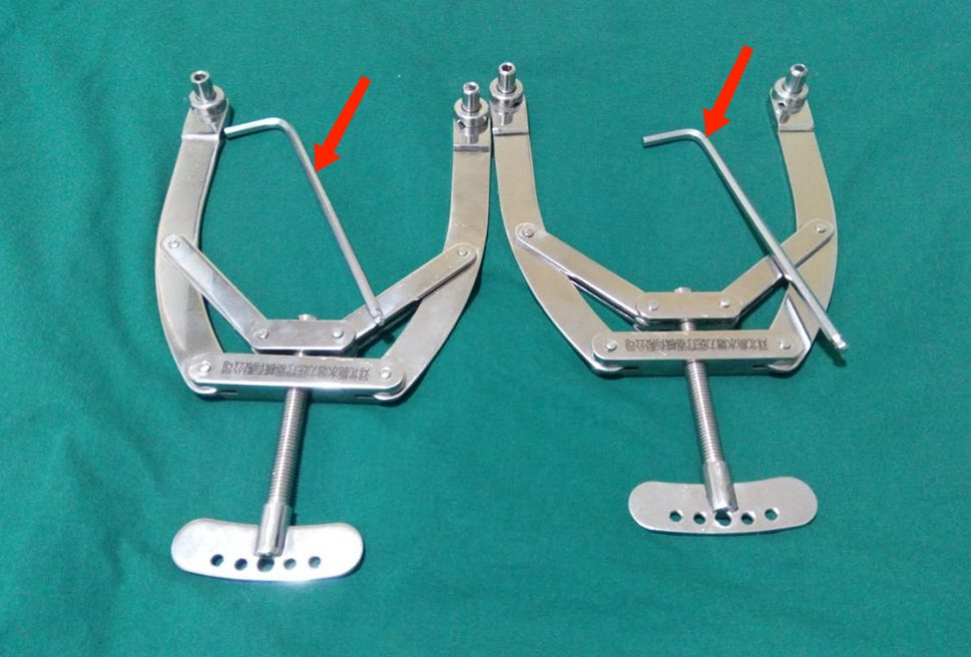
Wu’s Tension Traction Bows with matching hexagonal screwdrivers (marked with arrows).

2.0 mm diameter Kirschner wires assembled with Wu’s Tension Traction Bows were used in Group 1 (Fig. 2), and 3.5 mm diameter Steinmann pins assembled by Bohler’s traction bows were used in Group 2. The main traction procedures were basically the same and were as follows: the medial malleolus, posterior tip of calcaneus, and tibiotalar joint were marked as the superficial landmarks. Firstly, we drew a line from medial malleolar colliculus to posterior tip of calcaneus, so the second trisection point near the posterior tip of calcaneus was selected as the entry point. Secondly, 1% lidocaine was used to performed local infiltration anesthesia after disinfection with iodine povidone. Thirdly, we kept the ankle in dorsiflexion at 90º, and inserted the traction pin horizontally with a high-speed handheld electric drill from medial to lateral until the length of the traction pin was basically equal between two sides. Then the operation area was re-sterilized and dressed. Finally, after assembling with the traction bow, the traction weight approximating to 1/12 of the patient’s body weight was applied, and the traction alignment was adjusted appropriately according to the axis of the affected extremities.

**Figure 3:**
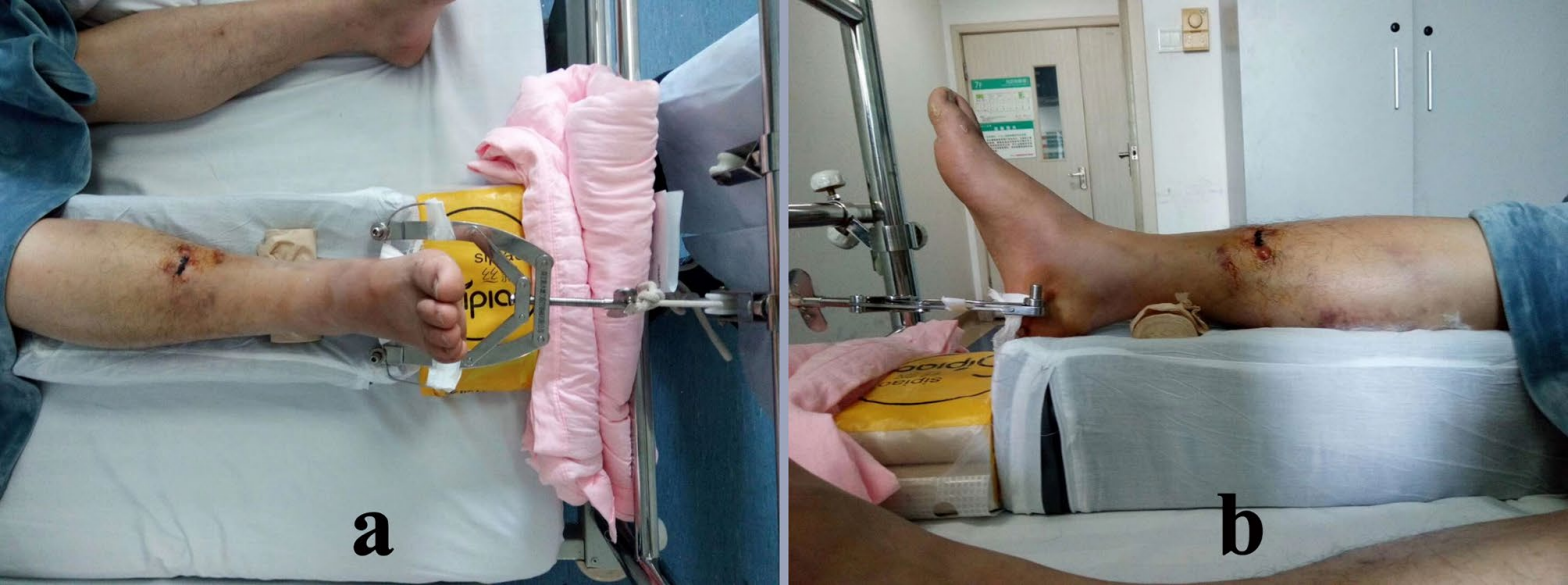
Application of Wu’s Tension Traction Bow assembled with a 2.0 mm diameter Kirschner wire in preoperative calcaneal skeletal traction for a patient with right tibia shaft fracture. Both ends of the Kirschner wires were bend and wrapped to prevent unexpected injury. (**a**) Top view. (**b**) Left lateral view.

**Figure 4:**
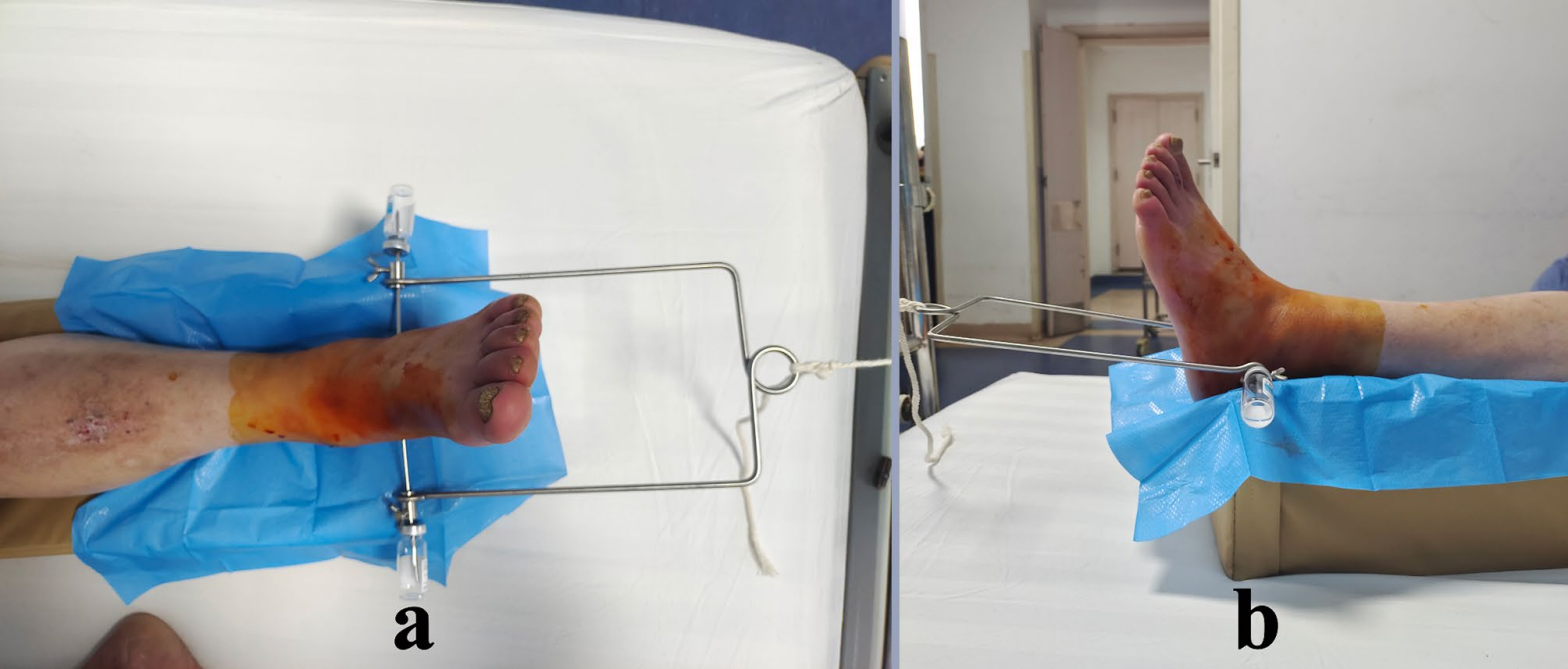
Application of Bohler’s Traction Bow assembled with 3.5 mm diameter Steinmann pins in preoperative calcaneal skeletal traction for a patient with left Pilon fracture. Since Steinmann pins were significantly more resistant to bending compared to Kirschner wires, a vial was slid onto each end of the Steinmann pin as an alternative solution to prevent unexpected injury. (**a**) Top view. (**b**) Left lateral view.

For optimum tension force in Wu’s tension bows, we recommended rotating the end knob clockwise until encountering strong resistance, to minimize the likelihood of bending the Kirschner wire while applying effective traction. Additionally, to prevent injury by sharp ends of traction pins, it was advised to bend both ends of the Kirschner wires and wrap them with tape, as shown in Fig. 3. However, considering robust Steinmann pins were resistant to bending, the alternative solution was to slide a vial onto each end of the pin, as shown in Fig. 4.

After completion of anesthesia on the day of surgery, traction appliances were removed. The first step involved disassembling the traction bow, followed by sterilizing the traction pin and the skin around the entry and exit points. For the intentionally bent Kirschner wires, the lateral or medial part was cut off as close to the skin as possible using a wire cutter. Subsequently, the wires were pulled out from the other side. In the case of Steinmann pins, which were difficult to cut, we pulled them out directly from either the lateral or medial side after strict sterilization. Following the removal of traction pins, the regional skin was re-sterilized, regardless of whether bleeding occurred. In instances where bleeding did occur, we applied pressure bandages and compression for five minutes. If bleeding persisted, small cotton balls were used to cover the wounds, followed by temporary bandaging using transparent medical plastic sheeting to prevent delays in subsequent operation procedures. At the end of the surgical procedures, the traction wounds were re-sterilized and dressed without suturing, and the same steps were repeated during daily ward rounds until the wounds were completely healed.

### Data collection

Patient demographic characteristics, including age, gender, cause of injury, fracture location, AO classification, and affected side, were obtained from the PDAS database. The cause of injury reflects the trauma energy, and AO fracture classification reflects the severity of the fracture. In both Group 1 and Group 2, the weight of traction, incidence and length of post-traction bleeding, effects on swelling reduction, length of preoperative traction, Visual Analogue Scale (VAS) pain score before implement of traction as well as the first 3 days after traction, size of traction wounds, and time from removal of traction appliances to completely healing of wounds were collected and then compared.

The severity of limb swelling was divided into 4 grades^3^. Degree 1: no obvious swelling and no abnormal changes compared to the normal limb; Degree 2: regional skin gets tighter than normal part, but dermatoglyph is still present. Degree 3: regional skin gets obviously tighter than normal part, and dermatoglyph disappears, but without tension blisters. Degree 4: regional skin gets severely tense and even shiny, dermatoglyph disappears, the superficial temperature gets noticeably higher than normal part, scattered tension blisters appear, localized pain is evident. On such basis, Swelling reduction was then assessed according to the time consumed from the date of traction implementation to the time when limb swelling reached Degree 2. Detailed criteria^3^ are as follows: (1) Markedly effective: < 3 days; (2) Effective: 3–6 days; (3) Fair: > 6 days. Both effective and markedly effective cases were used to calculate the overall effective rate.

### Statistical analysis

SPSS 26.0 (SPSS Inc., Chicago, IL, USA) was used for data processing. Independent t-tests or Mann–Whitney U tests were used for the comparison of continuous variables. Differences in categorical variables were determined using the chi-square test. A value of *P* < 0.05 was considered statistically significant.

## Results

A total of 65 patients were included, with a mean age of 39.1 ± 11.5 years, consisting of 52 males (80.0%) and 13 females (20.0%). Patients were divided into two groups based on the traction appliances used. Group 1 comprised 36 patients treated with 2.0 mm diameter Kirschner wires assembled with Wu’s Tension Traction Bows, while Group 2 consisted of 29 patients treated with 3.5 mm diameter Steinmann pins assembled with Bohler’s traction bows. The mean ages in Group 1 and Group 2 were 38.3 ± 12.1 years and 40.1 ± 10.9 years, respectively. Gender distribution (*P* = 0.454) and affected sides (*P* = 0.975) did not significantly differ between the two groups. The most common causes of injuries in Group 1 were traffic accidents (27.8%), falling from heights (44.4%), and sprains from walking or running (27.8%). In contrast, Group 2 reported traffic accidents (27.6%), falling from heights (37.9%), and walking or running sprains (34.5%) as major causes. In terms of fracture location within the groups, Group 1 displayed 41.7% proximal, 33.3% middle, and 25.0% distal tibia fractures, while Group 2 showed 41.4% proximal, 24.1% middle, and 34.5% distal tibia fractures. Detailed patient characteristics are provided in Table 1. Comparative analysis revealed no significant statistical differences in age, gender, causes of injuries, fracture location, and affected side between the two groups (Table 1, all *P* > 0.05), indicating their comparability.

In this study, the fracture locations were distributed over the full length of the tibia, leading to the use of the AO classification system to represent fracture severity. The detailed characteristics of fracture classification in both groups are presented in Table 2. The analysis revealed no statistically significant differences, thus strengthening the comparability of the two groups.

**Table 2:** Details of fracture classification.

	Group 1 (n = 36)	Group 2 (n = 29)	*P* value*
AO classification			0.958
41B	3	1	
41C	2	3	
42A	3	2	
42B	1	2	
42C	2	1	
43B	3	1	
43C	2	3	
44B	11	8	
44C	9	8	

*A value of *P* < 0.05 was considered to be statistically significant.

In both groups, no instances of traction pins loosening, breakage, or iatrogenic calcaneus fractures were observed. The average traction force exhibited no significant difference between the two groups, measuring 5.94 ± 0.65 kg in Group 1 and 6.05 ± 0.65 kg in Group 2 (*P* = 0.512), suggesting that thinner traction pins could withstand an equivalent traction force to thicker pins. Group 1 showed a lower incidence of post-traction bleeding with 4 cases (11.11%) experiencing bleeding for an average of 3.75 ± 0.96 days, in contrast to Group 2 where 9 cases (31.03%) had bleeding lasting an average of 5.67 ± 1.41 days, both findings being statistically significant (*P* < 0.05). Significantly fewer cases of bleeding and a shorter bleeding duration were associated with the use of 2.0 diameter Kirschner wires as traction pins. Regarding the effects on swelling reduction, Group 1 saw 6 cases classified as markedly effective, 24 as effective, and 6 as fair, compared to Group 2 where 2 cases were markedly effective, 21 were effective, and 6 were fair; however, no statistical difference was observed (*P* = 0.481) between the two groups. Similarly, no statistical significance was found between Group 1 and Group 2 in terms of the length of preoperative traction, which lasted for 5.80 ± 1.43 days and 6.07 ± 1.41 days, respectively. While the VAS pain score before skeletal traction and on the first two post-traction days did not differ significantly between Group 1 and Group 2 (*P* > 0.05), it was notably lower in Group 1 on the third day after traction (2.81 ± 0.71 compared to 3.17 ± 0.60 in Group 2, *P* = 0.030). Additionally, all traction wounds in both groups healed completely without infection. The average diameter of traction wounds was 2.85 ± 0.44 mm in Group 1 and 3.91 ± 0.46 mm in Group 2, showing a significant difference (*P* < 0.001). The mean time from removal of traction appliances to complete wound healing was 4.97 ± 1.52 days in Group 1 and 5.83 ± 1.58 days in Group 2, with a statistically significant variance (*P* < 0.05). This suggests that thinner traction pins led to smaller wounds that were more likely to heal promptly post-pin removal. Additional details can be found in Table 3, while Figs. 3 and 4 depict typical cases for each group.

**Table 3:** Outcomes of interventions.

Outcomes	Group 1 (n = 36)	Group 2 (n = 29)	*P* value*
Weight of traction (kg)	5.94 ± 0.65	6.05 ± 0.65	0.512
Post-traction bleeding			
Incidence	11.11% (4/36)	31.03% (9/29)	0.046**
Bleeding time	3.75 ± 0.96	5.67 ± 1.41	0.033**
Reduction of swelling			
Markedly effective	6 (16.67%)	2 (6.90%)	0.481
Effective	24 (66.67%)	21 (72.41%)	
Fair	6 (16.67%)	6 (20.69%)	
Length of traction	5.80 ± 1.43	6.07 ± 1.41	0.461
VAS pain score			
Before traction	7.22 ± 0.87	7.41 ± 0.91	0.389
After traction			
1st day	5.42 ± 0.87	5.17 ± 0.89	0.271
2nd day	4.00 ± 0.99	3.66 ± 0.67	0.113
3rd day	2.81 ± 0.71	3.17 ± 0.60	0.030**
Size of wounds	2.85 ± 0.44	3.91 ± 0.46	< 0.001**
Length of wound healing	4.97 ± 1.52	5.83 ± 1.58	0.030**

*A value of *P* < 0.05 was considered to be statistically significant.

**Difference was statistically significant.

## Discussion

Our study illustrates the feasibility and safety of utilizing 2.0 mm diameter Kirschner wires in conjunction with Wu’s Tension Traction Bows for calcaneal skeletal traction. When compared to the conventional method of utilizing 3.5 mm diameter Steinmann pins with Bohler’s traction bows, the thinner Kirschner wires combined with Wu’s Tension Traction Bows have been proven, in this study, to be sufficient in bearing the required weight of traction, alleviating pain, and accelerating swelling reduction. Furthermore, our findings indicate also shows a lower incidence of post-traction bleeding, a shorter duration of bleeding, and smaller traction wounds that heal more quickly.

Many fractures of lower limbs, such as tibial plateau fractures, pilon fractures, and severely displaced ankle fractures combined with or without ankle dislocation, often require preoperative temporary stabilization to be followed by a second-stage internal fixation after the regional soft tissue injuries have healed. Various preoperative temporary stabilization methods are typically employed, including plaster casts, skin traction, skeletal traction, and external fixation. Several studies have demonstrated that these management techniques can significantly enhance patients' prognosis^4,6–12^. While previous studies have predominantly focused on evaluating the clinical impact of preoperative temporary external fixation, limited research has directly compared the effectiveness of skin traction, skeletal traction, and external fixation^3,4,13,14^. Moreover, studies examining the differences in efficacy among different diameter traction pins in skeletal traction are scarce. This gap in the literature highlights the novelty of our study, as it may be among the first to explore and compare the outcomes of different traction technique and appliance in lower limb fractures.

Skeletal traction, an essential emergency management technique for patients with lower limb fractures, plays a crucial role in correcting shortening and angulation deformities resulting from fractures. It is also effective in relieving pain and swelling, thereby promoting the recovery of soft tissue injuries^1,15^. Comparatively, preoperative skeletal traction offers greater advantages over plaster casts. Firstly, it has been documented to ease the intraoperative fracture reduction process by rectifying deformities and maintaining the limb’s length, thereby facilitating a smoother surgery^16–18^; Secondly, unlike cast immobilization that wraps the affected limb, skeletal traction allows exposure of the injured area, facilitating the cold therapy to accelerate the reduction of swelling during the acute traumatic period. This exposure also enables real-time observation and evaluation of soft tissue recovery and permits prompt adjustments to traction alignment for correcting limb deformities as needed.

Skin traction is another preoperative stabilization technique that can be utilized as a provisional alternative to skeletal traction in various scenarios where the latter is impractical, such as in cases involving fractures or open soft tissue injuries near common skeletal traction entry points, patients with confirmed hypocoagulation, and settings with limited medical resources. Despite skin traction appearing less invasive and requiring less specialized skills compared to skeletal traction, its clinical outcomes have not proven to be as promising as anticipated^14,19–22^. Prolonged pressure associated with skin traction may lead to local soft tissue necrosis, exacerbate swelling, and possibly culminate in soft tissue infections. Furthermore, the marginal improvements in pain reduction and the pronounced discomfort related to traction have contributed to patients' unfavorable subjective experiences with skin traction. Daniel’s study^13^ showed that preoperative skeletal traction for patients with femoral fractures could be conductive to reduce limbs shortening, and be particularly effective in pain relieving, with VAS score decreasing from 7 in most patients to 1 or 2 after traction. This finding was consistent with our study, which showed that the majority of patients had a VAS score of no more than 3 on the 3rd day after skeletal traction. However, Daniel’s study^13^ reported a pin tract infection rate of 23.3%, whereas no infection related to traction pins was observed in our study. The possible explanation for the difference was that they used 4.5 mm diameter threaded Steinmann pins for skeletal traction, which were much larger in diameter than those used in our study. Additionally, although the threads on traction pins could probably increase the friction between traction pins and bone, which was beneficial to enhance the stability of skeletal traction, such pins could also cause more damage to both soft tissue and bone during insertion, subsequently resulting in a higher incidence of pin tract infection. However, our study has already showed that no significant differences were found either 2.0 mm diameter Kirschner wires or 3.5 mm diameter Steinmann pins used, in terms of traction weight, effectiveness of swelling reduction, length of preoperative traction duration and pain relief, but lower incidence and shorter duration of post-traction bleeding, as well as faster wounds healing, were definitely observed with the use of thinner traction pins. Given our study’s validation of the safety and efficacy of 2.0 mm diameter Kirschner wires for skeletal traction, and considering the elevated rates of pin tract infections reported with larger diameter traction pins in other studies^3,13^, in which 4.0 mm or lager diameter traction pins were used^3,13^, we advocate for the use of 2.0 mm diameter Kirschner wires in skeletal traction procedures.

In the past decades, with the advancement of medical technology and the increasing availability of medical resources, the utilization of external fixators in the initial treatment of fractures has been on the rise. However, despite satisfied outcomes confirmed by a large number of studies^23–26^, especially in the management of open fractures^12,27–29^, the widespread adoption of external fixators has been hindered by the significant financial costs and the requirement for a high level of medical expertise. The application of external fixators necessitates implementation under general or spinal anesthesia by experienced surgeons with the support of at least one assistant, leading to escalated medical expenses. In contrast, skeletal traction presents itself as a relatively simple and cost-effective alternative for preoperative stabilization. A less experienced surgeon can single-handedly carry out skeletal traction at the patient’s bedside under local anesthesia, incurring minimal costs compared to the substantial expenses associated with external fixation surgery. Wen et al.^3^ demonstrated the effectiveness of modified external fixation and calcaneal traction in the first-stage management of Ruedi-Allgower type II/III Pilon fractures. The study indicated that the overall rate of swelling reduction in the calcaneal traction group was 60.0%, while in our own investigation, the rates were 83.3% with 2.0 mm diameter Kirschner wires and 79.3% with 3.5 mm diameter Steinmann pins. This variance in outcomes could potentially be attributed to different sample characteristics of this two studies. In Wen’s research, all participants had Ruedi-Allgower type II/III Pilon fractures resulting from high-energy traumas, extending to the metaphysis and often accompanied by severe soft tissue damage, leading to more pronounced swelling and prolonged relief times. Additionally, 4 patients (13.3%) in Wen’s study who underwent skeletal traction developed pin tract infections^3^, potentially linked to the use of 4.0 mm diameter traction pins. Gao et al.^4^ also highlighted the effectiveness of calcaneal skeletal traction as a temporary stabilization technique in managing type C Pilon fractures in the initial stages, highlighting its simplicity compared to external fixators. In summary, when external fixation is not feasible, skeletal traction stands out as a commendable alternative. Based on our findings, we advocate for the adoption of 2.0 mm diameter Kirschner wires combined with Wu’s Tension Traction Bows for calcaneal skeletal traction, as this approach proves to be less invasive, facilitates faster recovery, and yields satisfactory traction outcomes.

However, several limitations are noteworthy in our study. Firstly, our study was retrospective and involved a relatively small sample size, rather than a prospective randomized controlled trial. Secondly, the assessment of swelling reduction relied on subjective measures; future studies should consider incorporating more objective swelling evaluation methods. Thirdly, limitations in our medical imaging equipment prevented us from obtaining the radiograph under skeletal traction, thus hindering the objective evaluation of skeletal traction’s effectiveness in correcting limb shortening and angulation deformity. Furthermore, the exclusion of patients with comorbidities like diabetes mellitus and grade 2 or higher hypertension may have reduced the study’s generalizability. For future research, a prospective study could investigate the traction device’s efficacy and safety in a population with complex comorbidities. Finally, though antithrombotic therapy was routinely given to all patients, its effects on coagulation function varied among individuals, potentially impacting post-traction bleeding incidence and duration.

## Conclusion

Our results show the feasibility and promising outcomes of 2.0 mm diameter Kirschner wires assembled with Wu’s Tension Traction Bows in calcaneal skeletal traction, which are also proven to be less invasive than 3.5 mm diameter Steinmann pins assembled with Bohler’s traction bows in our study. However, an urgent need exists for more reliable methods to assess reduction of limb swelling, as well as the maintenance of limb length and alignment, in order to enhance objectivity. Additionally, further demonstration of these findings necessitates the conduct of additional prospective randomized controlled studies with larger sample sizes. It is also crucial to consider individual variations in coagulation function when interpreting the results.

## Data Availability

The datasets used and/or analyzed during the current study are available from the corresponding author on reasonable request.
